# Digital-Human Public Community Care Integration for Chronic Pain in Low-Income Older Adults in a 6-Week Living Lab Setting: Quasi-Experimental Feasibility Study

**DOI:** 10.2196/85611

**Published:** 2026-04-20

**Authors:** Sunmi Song, Seo-Yeon Hwang, Hae-Young Kim, Junesun Kim

**Affiliations:** 1 College of Health Science, Department of Physical Therapy Korea University Seoul Republic of Korea; 2 Rehabilitation Science Program, Department of Health Sciences, Graduate School Korea University Seoul Republic of Korea; 3 Department of Health and Environmental Science, Undergraduate School, College of Health Science Korea University Seoul Republic of Korea; 4 BK21FOUR Program: Learning Health Systems, College of Health Science Korea University Seoul Republic of Korea; 5 Department of Public Health Sciences, Graduate School Korea University Seoul Republic of Korea; 6 Department of Health Policy and Management, College of Health Science Korea University Seoul Republic of Korea

**Keywords:** aging in place, community caregivers, depression, digital divide, digital health, health equity, pain management, quasi-experimental study, wearable sensors

## Abstract

**Background:**

Digital health technologies offer promising solutions for managing chronic pain and depression in older adults, yet low-income populations with limited digital literacy face substantial barriers to access. Community-based approaches that leverage existing care infrastructure may bridge this digital divide, but evidence remains limited on effective integration strategies for digitally excluded populations.

**Objective:**

We evaluated a multimodal digital health monitoring platform for this underserved population through 6-week living lab trials with 86 low-income Korean older adults (43 intervention and 43 age- and gender-matched controls) and 25 community caregivers.

**Methods:**

Participants were recruited offline from recipients of government-supported community care services. The platform integrated chatbot surveys, smartwatch monitoring, and motion sensors, generating personalized traffic-light alerts when health indicators deviated from individual baselines and triggering real-time caregiver notifications for persistent declines. Mobile apps for older adult users and caregivers, along with a centralized monitoring system for community center managers, were provided to the intervention group, while controls received standard community care. Pre- and posttest surveys assessed changes in pain-related functional limitations, depressive symptoms, sleep quality, and system usability through face-to-face assessments. Caregivers documented platform-triggered interventions.

**Results:**

Following attrition due to illness (n=3) and participation burden (n=4), we analyzed 1318 days of continuous monitoring data from 35 intervention participants (mean 37.7, SD 8.8 days per participant), with 77 participants (35 intervention and 42 control) completing pre-post assessments. Distinct patterns emerged in 24-hour heart rate profiles: participants experiencing higher-than-usual pain demonstrated elevated heart rates during early morning (5-8 AM) and late evening (10-11 PM) hours compared to their low-pain days. Multilevel modeling revealed significant within-person associations between digital biomarkers and symptoms. Heart rate variability (*P*=.02) and moderate physical activity (*P*=.05) were associated with same-day pain. Heart rate variability (*P*=.03) and long wake episodes during sleep (*P*=.03) predicted next-day pain. Shorter sleep duration (*P*=.03) and lower sleep efficiency (*P*=.05) were associated with same-day depressive symptoms. A significant group-by-time interaction was observed for pain-related functional limitations (*P*=.03): the intervention group maintained baseline levels while the comparison group reported increased functional limitations. Depressive symptoms, sleep quality, and platform usability did not show significant changes. Community caregivers successfully conducted 37 health decline–triggered interventions during regular service hours (25 hours per week), demonstrating integration of digital monitoring with existing care workflows.

**Conclusions:**

Integrating digital health monitoring within existing community care infrastructure may be a feasible approach to supporting vulnerable older adults. The intervention was associated with maintained pain-related functional limitations, while depressive symptoms and sleep quality showed no significant changes, possibly due to the 6-week duration. This feasibility study provides preliminary support for digital-human integration models to address health equity, though randomized controlled trials are needed to establish causal relationships and isolate active intervention components.

**Trial Registration:**

ClinicalTrials.gov NCT06270121; https://clinicaltrials.gov/study/NCT06270121

## Introduction

The global demographic shift toward population aging has created an urgent need for innovative health care solutions [1,2]. Digital health technologies offer unprecedented opportunities for continuous monitoring and personalized care [3,4], particularly valuable for older adults managing multiple chronic conditions, including pain and depression [5,6]. However, the populations who could benefit most from these innovations—low-income older adults with limited digital literacy [7,8]—face the greatest barriers to accessing them [2,9]. This digital health divide is especially pronounced among socially isolated older adults, who experience higher rates of depression and chronic pain while lacking the support networks essential for technology adoption and health management [10,11].

Digital phenotyping—the continuous collection of behavioral and physiological data through digital devices—has shown promise in detecting and managing pain and mental health conditions in various populations [12-17]. Studies have demonstrated associations between wearable sensor data and depression symptoms, with heart rate variability (HRV), sleep patterns, and physical activity (PA) serving as reliable indicators of mental health status. Similarly, smartphone-based assessments have successfully captured daily fluctuations in pain and functional limitations among adults with chronic conditions. Recent evidence suggests that continuous monitoring can detect symptom changes before clinical deterioration, enabling timely interventions [3,18-20]. However, most digital phenotyping research has focused on younger, digitally literate populations, leaving a critical gap in understanding how these technologies might benefit older adults who face both high disease burden and significant technology barriers [5,21].

Multiple factors contribute to the digital exclusion of older adults. Age-related sensory and cognitive changes create difficulties in learning new technologies [22], while low income limits access to devices and internet connectivity [7,23,24]. Social isolation compounds these challenges, as older adults lack the informal support networks that facilitate technology learning [2]. Previous digital health interventions for older adults have shown mixed results, often failing due to high dropout rates and usability issues [25,26]. Even well-designed platforms struggle when they require direct interaction from users with limited digital skills and cognitive impairments [27,28]. These failures highlight a fundamental design flaw: most digital health solutions assume a level of digital literacy that many vulnerable older adults do not possess, perpetuating rather than addressing health inequities.

Emerging evidence suggests that community-based approaches may overcome these barriers. Studies have shown improved outcomes when digital interventions are delivered through trusted intermediaries such as nurses or trained caregivers [29-31]. Living lab methodologies, which embed technology development within users’ natural environments, have demonstrated success in creating solutions that are feasible and sustainable in real-world settings [29,32]. In many countries, existing community care infrastructure already provides regular contact between formal caregivers and vulnerable older adults. These established relationships and routine visits represent an untapped opportunity for digital health delivery. By positioning caregivers as technology intermediaries rather than expecting direct use by older adults, digital health platforms could leverage existing human infrastructure to bridge the digital divide.

This study addresses this critical gap by evaluating the feasibility and preliminary effectiveness of integrating multimodal digital health monitoring within existing community care infrastructure for low-income, socially isolated older adults in South Korea. Using a 6-week living lab quasi-experimental design with an intervention and matched comparison group, we aim to answer the following three research questions: (1) Can digital biomarkers derived from continuous monitoring predict daily variations in pain and depression among older adults? (2) Does digital monitoring integrated with existing care services improve functional health outcomes compared to usual care? (3) Is it feasible to integrate digital monitoring within existing community care infrastructure without disrupting established workflows? We hypothesized that daily fluctuations in HRV, sleep quality, and PA would be associated with same-day and next-day pain and depressive symptoms (hypothesis 1 [H1]); participants receiving the integrated intervention would demonstrate improved or maintained functional health outcomes compared to those receiving usual care alone (hypothesis 2 [H2]); and community caregivers would successfully integrate platform-generated health alerts into their existing care workflows, enabling timely interventions for detected health declines without requiring additional work hours or service costs (hypothesis 3 [H3]. This quasi-experimental design would allow feasibility evaluation of an integrated digital-human care model rather than isolated digital platform effects.

**Figure 1:**
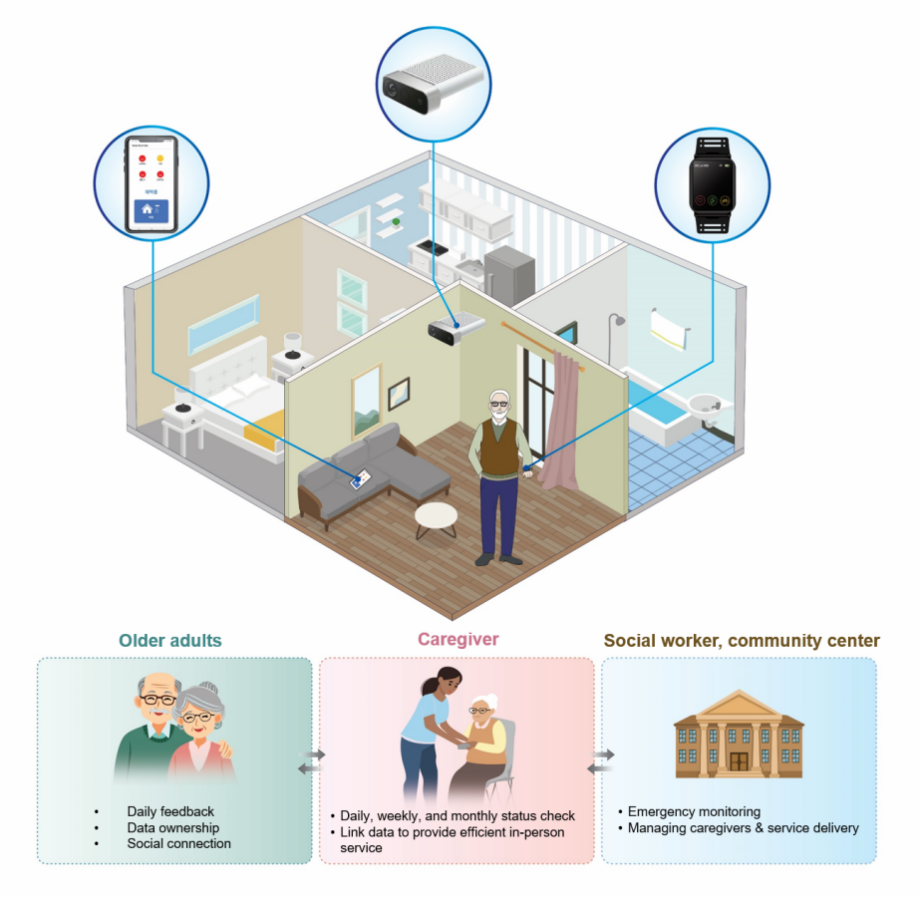
Community-mediated digital health platform architecture for vulnerable older adults. The 3-tiered service delivery system enables comprehensive health monitoring and care coordination. At the individual level, older adults in their homes interact with smartphone apps, smartwatches, and motion sensors for continuous health monitoring. When personalized algorithms detect health deterioration signals, assigned community caregivers provide targeted support through phone calls or home visits. For severe health declines or emergencies requiring coordinated response, the system escalates to the community center for comprehensive intervention. This hierarchical architecture ensures appropriate care intensity while maintaining older adults’ independence in their home environment.

**Figure 2:**
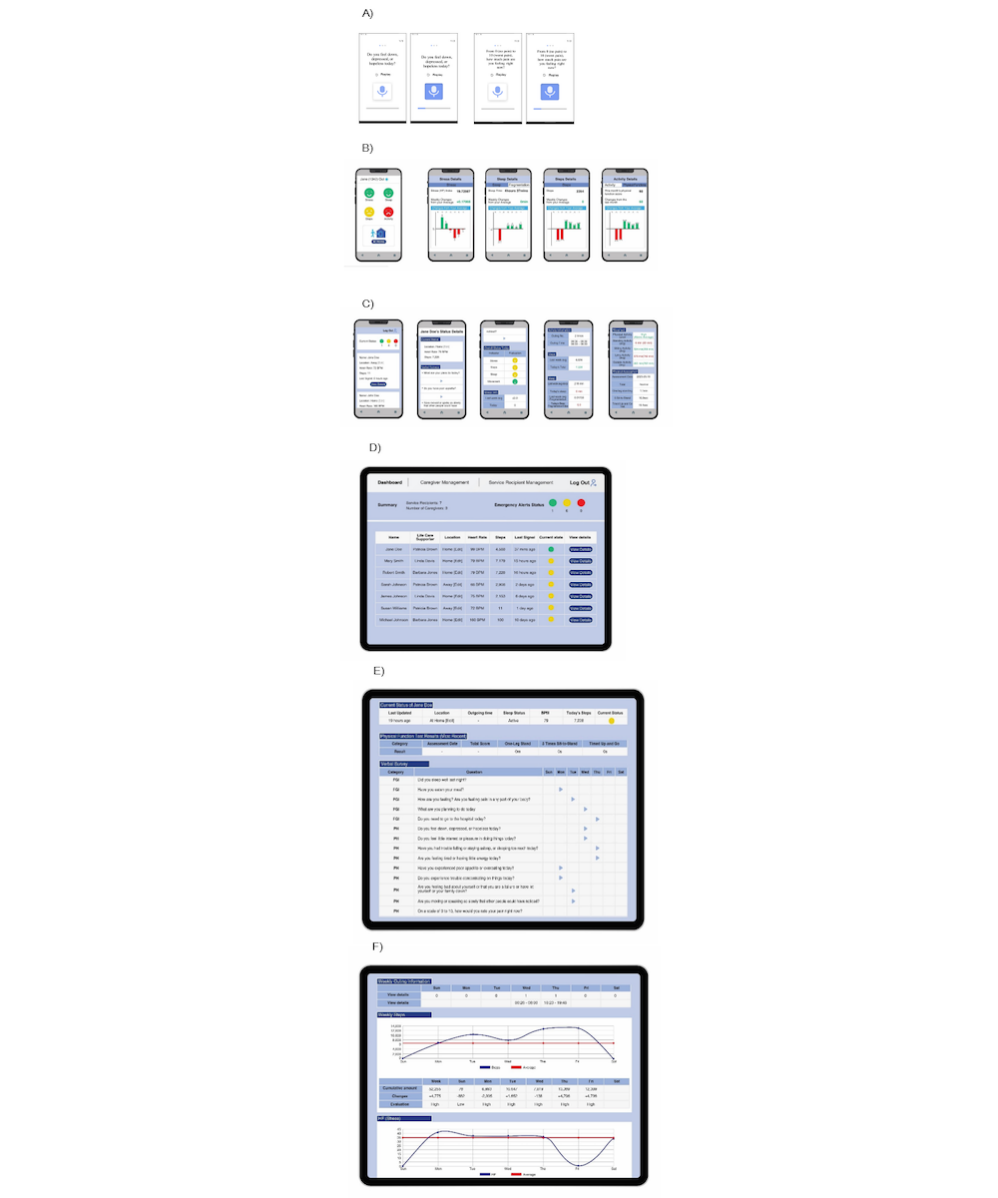
Platform interfaces demonstrating the traffic-light feedback system. (A) User application showing morning chatbot interaction for pain and depressive symptom reporting. (B) Personalized health status display using intuitive traffic-light indicators (green: improvement; yellow: stable; and red: decline from baseline) and detailed weekly pattern views for 4 health domains (stress, sleep, steps, and activity). (C) Caregiver dashboard displaying multiple recipients’ health status. (D-F) Administrative interface for center managers showing aggregate health trends, alert history, and intervention tracking. This design simplifies complex health data into actionable information for major stakeholders. A higher resolution version of this image is available in Multimedia Appendix 1.

Based on previous formative and pilot study [33], our approach integrates continuous digital phenotyping through smartwatches and daily chatbot surveys with existing community care services, where caregivers serve as technology intermediaries (Figure 1). The platform’s traffic-light feedback system simplifies complex health data into actionable information for both users and caregivers (Figure 2).

## Methods

### Overview

This study represents the culmination of a multiphase research program aimed at developing and evaluating a community-integrated digital health monitoring platform for socially vulnerable older adults. The development process began with formative research involving surveys (n=99 older adults) and focused group interviews with older adults (n=16), community caregivers (n=12), and social workers (n=3) to identify primary health concerns, understand community care workflows, and determine essential platform features. This informed the platform design, which was subsequently refined through an informal prepilot test with study staff (n=6) and a 6-week single-arm living lab pilot study (n=31) to establish feasibility and optimize functionality, which was previously reported [33].

Building on these foundational phases, this study used a 6-week quasi-experimental design with matched comparison groups to evaluate the feasibility and preliminary effectiveness of integrating digital monitoring within existing community care services. The evaluation encompassed not only the digital monitoring components but also the integration with in-person community care services and the institutional emergency alert system that enables coordinated responses from caregivers and center managers when health decline is detected. The research was conducted at a community senior welfare center in Seoul, South Korea, from October to December 2023, following formative and pilot phases that spanned the COVID-19 pandemic period (2021-2022). This iterative development approach—progressing from needs assessment through pilot testing to quasi-experimental evaluation—ensured the platform was both technically robust and contextually appropriate for the target population of low-income older adults with limited digital literacy. This iterative process was guided by a participatory, user-centered approach that engaged older adult participants, community caregivers, and welfare center administrators during and after the pandemic period. Their input informed not only platform design but also key study design decisions, including the adoption of a matched comparison rather than a randomized design, which stakeholders collectively identified as essential for ethical engagement that respects the right of choice and dignity of this vulnerable population.

Older adult participants received the Korean Government–supported Community Care Service that was provided free of charge: twice-weekly phone calls and weekly home visits from assigned caregivers, with additional follow-up services provided for acute illness episodes. Caregiver work hours are regulated at 25 hours per week under the service protocol, and poststudy caregiver surveys confirmed adherence to this standardized schedule in the intervention group without overtime work. The platform-triggered interventions in the intervention group aimed to facilitate earlier detection of health decline events; caregivers were briefly trained to use the platform to identify emerging health issues through digital alerts before these became apparent during scheduled contacts or escalated to acute episodes.

### Ethical Considerations

This study was conducted in accordance with the Declaration of Helsinki and approved by the Institutional Review Boards of Korea University (approval number KUIRB-2021-0324) and Sahmyook University (approval number SYU 2022-06-003-001). Written informed consent was obtained from all individual participants included in the study prior to enrollment. Safety monitoring was conducted through community caregivers who served as technology intermediaries. Caregivers received daily health status updates via the traffic-light dashboard and responded to health deterioration alerts with phone calls or in-person visits, enabling continuous safety monitoring and timely response to detected health concerns. Privacy was protected through secure data transmission and restricted access to participant health information, with only assigned caregivers, a community center manager, and authorized research staff permitted to view individual health data.

### Recruitment

Participants were identified through the Community Care service database at a municipal senior welfare facility in Seoul. Community caregivers facilitated recruitment by identifying eligible older adults from their existing caseloads and arranging initial contact with the research team.

Inclusion criteria comprised (1) age of 65 years or older, (2) living alone with low income qualifying for government-supported care services, (3) currently receiving biweekly phone calls and weekly home visits through the Community Care service, (4) possession of an Android smartphone compatible with the study application, and (5) capacity to provide informed consent. Individuals were excluded if they had cognitive impairment preventing study participation, severe sensory impairments interfering with technology use, cohabitation with others (due to motion sensor limitations), or medical conditions requiring intensive care.

The intervention group (n=43) consisted of older adults whose caregivers agreed to use the digital platform, while the comparison group (n=43) included age- (±3 years), gender-, and residential area–matched older adults whose caregivers maintained standard care practices. A total of 25 community caregivers participated as technology intermediaries for the intervention group.

### Digital Health Monitoring Platform

The platform architecture integrated 3 synchronized applications: a user interface providing personalized health feedback, a caregiver dashboard for monitoring multiple recipients, and an administrative system for center managers (Figure 1). Daily health indicators were visualized using an intuitive traffic-light system (green: better than baseline, yellow: similar to baseline, red: worse than baseline), calculated from each participant’s first-week average (Figure 2). Detailed technical specifications and user interface designs of the platform have been previously described in our pilot study [33]. The results from the motion sensing camera, which was integrated with experimental sessions outside the living lab, are reported separately from this study [34].

Technical components included a chatbot-enabled smartphone app for daily surveys, continuous physiological monitoring via Fitbit Sense smartwatches, and Azure Kinect motion sensors for home-based activity detection. While motion-sensing cameras were deployed as part of the platform infrastructure, their biomechanical data were analyzed in a concurrent validation study and are presented separately [34].

Physiological measures were derived from the Fitbit Sense smartwatch, a consumer-grade device. While Fitbit devices have demonstrated acceptable validity for PA, resting heart rate, and irregular heart rhythms compared to research or clinical-grade sensors [35,36], heart rate measurements during active periods and sleep staging algorithms show greater variability against polysomnography gold standards, particularly in populations with sleep disorders or arrhythmias [37,38]. Our older adult participants’ high prevalence of chronic conditions may have introduced measurement noise. Thus, the use of a consumer-grade sensor in this study may have attenuated observed associations or introduced measurement error.

### Intervention Protocol

Following informed consent and baseline assessment, research staff and caregivers conducted home visits to install applications and provide initial training. Participants engaged with the chatbot each morning, responding to 2 randomly selected Patient Health Questionnaire-9 (PHQ-9) items for depressive symptom screening and rating pain levels (0-10 visual analog scale [VAS]). Continuous smartwatch monitoring captured HRV, sleep patterns, and PA throughout the study period.

Community caregivers received daily updates on participants’ health status through the traffic-light system displayed on their dashboard. When participants exhibited multiple red indicators (≥2 domains) for 2 or more consecutive days, caregivers were instructed to conduct follow-up interventions via phone calls or in-person visits based on severity assessment. All caregiver-initiated interventions triggered by health decline signals were systematically documented and analyzed (Figure S1 in Multimedia Appendix 2 for examples of health decline signals). Table S3 in Multimedia Appendix 2 shows the records of the community caregiver’s follow-up reports after detecting health decline signals. This quasi-experimental study with both an intervention and a comparison group has been registered on ClinicalTrials.gov (NCT06270121).

### Daily Digital Measures of Pain and Depression

Intervention participants completed daily assessments via the chatbot interface that was developed in our pilot study [33]. For depressive symptom screening, 2 items were randomly selected from the PHQ-9 questionnaire and presented in a yes/no format each day. Participants who responded “yes” to one or both items were coded as experiencing depressive symptoms for that day. Pain intensity was assessed using a VAS (0-10, where 0=no pain, 10=worst imaginable pain). Response rates and patterns were analyzed as engagement indicators.

### Digital Biomarker Measurements

Continuous physiological monitoring via Fitbit Sense smartwatches captured 3 domains of digital biomarkers throughout the 6-week intervention period, as previously described [33]. Sleep metrics included total sleep time, sleep fragmentation index (awakening frequency divided by total sleep time), number of long wake episodes (over 5 minutes of awakening), and sleep efficiency (total sleep time divided by time in bed), calculated using validated algorithms that applied 7 correction criteria to improve actigraphy-based sleep detection accuracy [39,40].

HRV was computed every 5 minutes using the open-source “Aura-healthcare” Python package [41], generating both time-domain indicators (SD of N-N intervals and root-mean-square of successive differences) and frequency-domain measures (high-frequency/low-frequency ratio) from R-R interval data [41,42].

PA levels were quantified through daily step counts and minutes of light, moderate, and intense activity, leveraging Fitbit’s validated 3-axis accelerometer algorithms [35,43,44]. These multidomain digital biomarkers provided comprehensive 24-hour physiological profiles for examining within-person associations with daily pain and depressive symptoms.

### Pre- and Postsurvey Measures

Primary and secondary outcomes were assessed through structured home visits at baseline, week 3 (midintervention), and week 6 (postintervention) by trained research assistants. Caregivers also participated in brief surveys at pretest and posttest.

#### Pain-Related Functional Limitations

The Korean version of the Western Ontario and McMaster Universities Osteoarthritis Index (WOMAC) was administered to assess pain-related functional impairment [45,46]. WOMAC comprises 24 items evaluating 3 dimensions: pain (5 items), stiffness (2 items), and physical function (17 items). Each item is scored on a 5-point Likert scale (0-4), with higher total scores indicating greater functional limitations.

#### Geriatric Depression Scale Short-Form

The 15-item Geriatric Depression Scale Short-Form (GDS-SF) was used to screen for depressive symptoms [47,48]. Each item requires a yes/no response, with scores ranging from 0 to 15. Clinical cutoffs indicate normal (0-4), mild depression (5-9), and severe depression (≥10). The GDS-SF has shown high sensitivity (92%) and specificity (89%) for detecting depression in Korean older adults.

#### Pittsburgh Sleep Quality Index

The Pittsburgh Sleep Quality Index (PSQI) assessed subjective sleep quality over the past month [49,50]. This 19-item questionnaire evaluates 7 components: subjective sleep quality, sleep latency, sleep duration, habitual sleep efficiency, sleep disturbances, use of sleep medications, and daytime dysfunction. Global PSQI scores range from 0 to 21, with scores >5 indicating poor sleep quality. The Korean PSQI has demonstrated good internal consistency (Cronbach α=0.84) and test-retest reliability (*r*=0.85).

#### Technology Usability

System usability was measured using a simplified System Usability Scale (SUS) adapted for cognitively impaired and older adults [51]. The scale contains 10 items rated on a 5-point scale (strongly disagree to strongly agree), yielding scores from 0 to 100. Higher scores indicate better perceived usability, with scores >68 considered above average. Preintervention SUS assessed general technology use, while postintervention SUS specifically evaluated the digital health platform. To assist participants unfamiliar with digital technology, examples (smartphones and hospital kiosks) were provided during baseline assessment.

In addition to the SUS, participants were asked about the frequency of platform use and the number of functions used during the midterm assessment. At postintervention, participants provided qualitative feedback for platform improvement, which research assistants documented verbatim. Community caregivers similarly completed the SUS and reported their usage frequency and range of functions used at the postintervention assessment.

#### Covariates

Covariates were determined a priori based on extant literature summarizing demographic and health risk factors for daily depressive symptoms of older adults [52,53]. In addition to the baseline levels of depression, demographic and health factors linked with depression (age, sex, and chronic health conditions) were used as covariates.

#### Caregiver Assessments

Caregivers participated in brief surveys at pretest about their demographic and job-related information, and at posttest about their usage of the caregiver app. Poststudy surveys assessed the SUS survey, caregiver platform engagement, including working hours compliance, usage frequencies, function use, and qualitative feedback on workflow integration and user experience.

### Statistical Analysis

After analyzing missing data and assessing the distributions of key variables, descriptive analyses were performed. Group comparisons between older adults with depression and without depression were conducted using *t* tests for continuous variables (eg, age, BMI, PSQI total score, daily depressive symptoms, wearable sensor assessments of HRV, sleep, and PA indicators), chi-square tests for categorical variables (eg, sex, education, income, chronic disease, and sleep disorder categories). Subsequently, bivariate correlations were explored between baseline depression and person mean variables (daily pain and depressive symptoms, and wearable sensor assessments) over 6 weeks.

Within-person associations between digital biomarkers and daily symptoms were examined using multilevel models (SAS/STAT version 9.4; SAS Institute Inc). Level 1 examined daily variations in symptoms and sensor metrics; level 2 incorporated participant characteristics. It allows for the use of participants as their own controls [54].

For pain as outcome, the model included daily depressive symptoms (binary coded: 0=no symptoms, 1=responded “yes” to at least 1 of 2 daily PHQ-9 items), sleep, HRV, and PA indices were examined as predictors of same-day and next-day pain levels. For depressive symptoms as outcome, the model included daily pain levels (VAS 0-10), sleep, HRV, and PA indices as predictors of same-day and next-day depressive symptoms using logistic multilevel models, given the binary nature of the outcome.

All level 1 predictors were person mean–centered, while level 2 covariates (age, sex, and chronic conditions) were grand mean-centered. This approach allows interpretation of estimates as within-person deviations from individual averages across the 6-week period. Bonferroni corrections were applied for multiple testing (α=.005).

The pretest and posttest results were assessed using a mixed model with time (pretest and posttest) at level 1, personal characteristics (covariates of age, gender, and chronic disease conditions) at level 2, and intervention group at level 3. All plots of results were drawn using the R software (version 4.3.1; R Foundation for Statistical Computing) ggplot2 package [55].

### Power Analysis

Simulation studies demonstrate that 35 participants with >30 days of continuous monitoring yields adequate statistical power for detecting within-person effects in multilevel models [56]. For pre-post comparisons, 72 participants (32 intervention and 40 comparison) completed end point assessments, providing 80% power to detect medium effect sizes (*d*=0.50) in group × time interactions. Although there is no prior study to estimate effect sizes from older adult populations, this is consistent with meta-analytic evidence from human-guided digital interventions for depression in general adult populations (Hedges *g*=0.52-0.63) [57], which represents the closest available benchmark for digital-human integration models. Given the modest sample size, findings should be interpreted as preliminary rather than definitive evidence of intervention effectiveness.

### Data Exclusion and Missing Data

Of 86 enrolled participants, 77 provided analyzable data (35 intervention and 42 comparison), with 9 exclusions due to withdrawal (n=4 participation burden), illness (n=2), relocation (n=1), or incomplete baseline data (n=2). The intervention group generated 1318 observation days (mean 37.7, SD 8.8 days per participant) for multilevel analyses.

Missing data analyses showed missing in daily surveys (455/1318, 34.5%), HRV (356/1318, 27%), sleep monitoring (625/1218, 47.4%), and PA (126/1318, 9.6%). Missing data primarily resulted from forgetting to wear devices or charging issues, rather than technical failures or dropout. Full information maximum likelihood estimation addressed missing data, a method shown to be robust against bias with partial missingness [58].

### Protocol Deviations

The trial registration (ClinicalTrials.gov NCT06270121) originally specified the Short-Form 12 health survey as the primary quality of life outcome. During implementation, we modified the primary outcome to the WOMAC to focus specifically on pain-related functional limitations, as baseline assessments revealed that 90% (70/73) of participants experienced chronic pain, making functional outcomes more clinically relevant and responsive to our 6-week intervention than generic quality of life measures. Additionally, the PSQI was added as a secondary outcome after initial data showed that 69.2% (54/78) of participants exceeded the clinical cutoff for sleep disturbances (PSQI >5), highlighting the importance of sleep assessment in understanding the pain-mood-sleep triad in this population.

## Results

### Participant Characteristics

**Figure 3:**
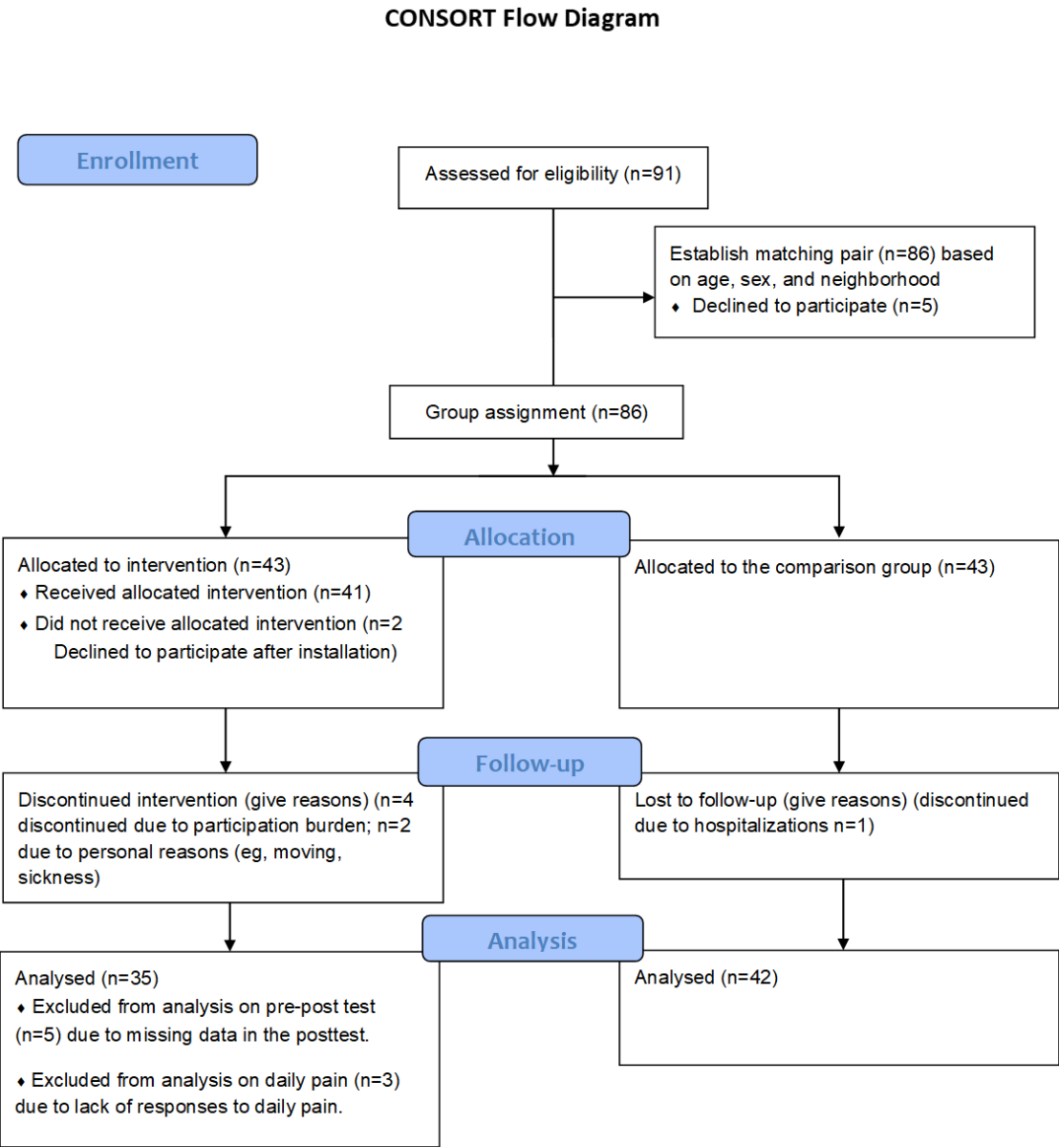
CONSORT (Consolidated Standards of Reporting Trials) flow diagram of participant recruitment and retention. Study enrollment occurred in October-November 2023 in Seoul, South Korea. Of 91 eligible older adults approached, 86 enrolled (43 matched pairs). The intervention group received digital health monitoring integrated with community care services; the comparison group received usual care. Attrition was primarily due to illness (n=3) or participation burden (n=4). Analysis includes 35 intervention and 42 comparison participants for pre-post outcomes, with 35 intervention participants contributing 1318 days of digital phenotyping data.

Between April and November 2023, we enrolled 78 low-income older adults living alone in Seoul, South Korea (35 intervention and 43 comparison) and 25 community caregivers. The digital health monitoring platform design and service delivery model are illustrated in Figures 1 and 2. Participant flow through the study is shown in Figure 3.

Baseline characteristics demonstrated comparable demographics and high disease burden across groups (Table 1). Mean age was 78 (SD 4.1) years, 85% (66/78) were women, and participants had limited education (mean 8.2, SD 4.7 years). Monthly income averaged KRW 900,000 (approximately US $670), confirming socioeconomic vulnerability. Participants reported an average of 4 chronic conditions, predominantly hypertension (46/78, 59%), diabetes mellitus (45/78, 58%), cataracts (43/78, 55%), and arthritis (25/78, 49%). Notably, 90% (70/78) experienced chronic pain lasting over 3 months, primarily affecting the back (50/78, 64%) and knees (39/78, 50%). Baseline assessments revealed mild depressive symptoms (mean GDS-SF score 4.3, SD 3.5), poor sleep quality (mean PSQI score 8.0, SD 3.8, exceeding the clinical cutoff of 5), and moderate pain-related functional limitations (mean WOMAC score 29.1, SD 19.0). Groups showed no significant baseline differences (all *P*>.05). Characteristics of caregivers and their platform usage were also reported in Table 2.

**Table 1:** Baseline characteristics of older adult participants demonstrate comparable demographics and high disease burden across groups.

Characteristics				Total (N=78)	Group assignments			*P* value
					Intervention group (n=35)	Comparison group (n=43)		
**Demographic characteristics**								
	Age (years), mean (SD)			78.03 (4.10)	78.34 (3.15)	77.77 (4.76)	.53	
	Sex: women, n (%)			66 (84.62)	28 (80)	38 (88.37)	.31	
	Years of education, mean (SD)			8.21 (4.70)	7.17 (4.39)	9.05 (4.83)	.08	
	**Monthly income (KRW^a^), n (%)**							
		Less than 500,000		17 (21.79)	8 (22.86)	9 (20.93)	.19	
		500,001-1,000,000		40 (51.28)	20 (57.14)	20 (46.51)	—^b^	
		1,000,001-1,500,000		9 (11.54)	5 (14.29)	4 (9.30)	—	
		1,500,001 or more		12 (15.38)	2 (5.71)	10 (23.26)	—	
	Currently employed			14 (18.18)	8 (22.86)	6 (14.29)	.33	
**Health characteristics, mean (SD)**								
	BMI			24.73 (3.53)	24.05 (3.06)	25.27 (3.81)	.14	
	Number of chronic diseases			4.24 (1.83)	4.31 (2.05)	4.18 (1.65)	.76	
	Baseline depressive symptoms (GDS-SF^c^, 0~15)			4.28 (3.53)	4.42 (3.66)	4.16 (3.46)	.74	
	Baseline sleep quality (PSQI^d^, 0~21)			8.02 (3.82)	8.06 (3.63)	8.00 (4.01)	.95	
	**Baseline pain status**							
		**Pain duration, n (%)**						
			Chronic pain >3 months	70 (89.74)	29 (82.86)	41 (95.35)	.07	
			Acute pain	4 (5.13)	4 (11.43)	0 (0)	—	
			No pain	4 (5.13)	2 (5.71)	2 (4.65)	—	
		**Pain locations, n (%)**						
			Back	50 (64.10)	24 (68.57)	26 (60.47)	.46	
			Knee	39 (50)	13 (37.14)	26 (60.46)	.04	
			Neck and shoulder	21 (26.92)	7 (20)	14 (32.56)	.21	
			Calf	17 (21.79)	11 (31.43)	6 (14)	.06	
			Others	34 (43.59)	16 (45.71)	18 (41.86)	.73	
			Baseline pain severity (WOMAC^e^, 0-100)	29.06 (18.96)	29.57 (19.88)	28.65 (18.41)	.83	

^a^1 KRW ≈ US $0.00074.

^b^Not available.

^c^GDS-SF: Geriatric Depression Scale Short-Form.

^d^PSQI: Pittsburgh Sleep Quality Index.

^e^WOMAC: Western Ontario and McMaster Universities Osteoarthritis Index.

**Table 2:** Characteristics and the platform usage of community caregivers.

Characteristics				Total (n=25)
**Demographics characteristics**				
	Age (years), mean (SD)		61.24 (10.1)	
	Sex (women), n (%)		25 (100)	
	**Education, n (%)**			
		Elementary school or less	2 (8)	
		Middle school	2 (8)	
		High school	6 (24)	
		College or above	13 (52)	
		Choose not to report	2 (8)	
	**Marital status, n (%)**			
		Married	17 (68)	
		Widowed	4 (16)	
		Divorced	3 (12)	
		Never married	1 (4)	
	**Job characteristics**			
		Work experiences (months), mean (SD)	63.56 (45.20)	
		Senior care provider (yes), n (%)	5 (20)	
		Number of care-providing older adults (persons), mean (SD)	10.96 (2.09)	
		Weekly hours of work during intervention, mean (SD)	25.4 (1.38)	
**Caregiver app usage^a^**				
	System Usability Scale (0-100) at posttest, mean (SD)		70.80 (16.67)	
	**Frequency of use, n (%)**			
		More than 2-3 times per day	5 (21.7)	
		2-3 times per day	11 (47.8)	
		Once a day	6 (24)	
		Once every 2-3 days	1 (4.3)	
	Number of functions used, mean (SD)		6 (3.92)	
	**Functions used, n (%)**			
		Listening to the voice recording of older adults	19 (82.6)	
		Steps of older adults	14 (60.9)	
		Stress levels of older adults	13 (56.5)	
		Activity at the home of older adults	12 (48)	
		Sleep of older adults	12 (52.2)	
		Emergency status	6 (27.3)	
		Etc (eg, weekly patterns or health status alerts)	14 (56.2)	

^a^2 caregivers were not able to participate in the posttest surveys due to schedule conflicts.

### Descriptive Results

**Figure 4:**
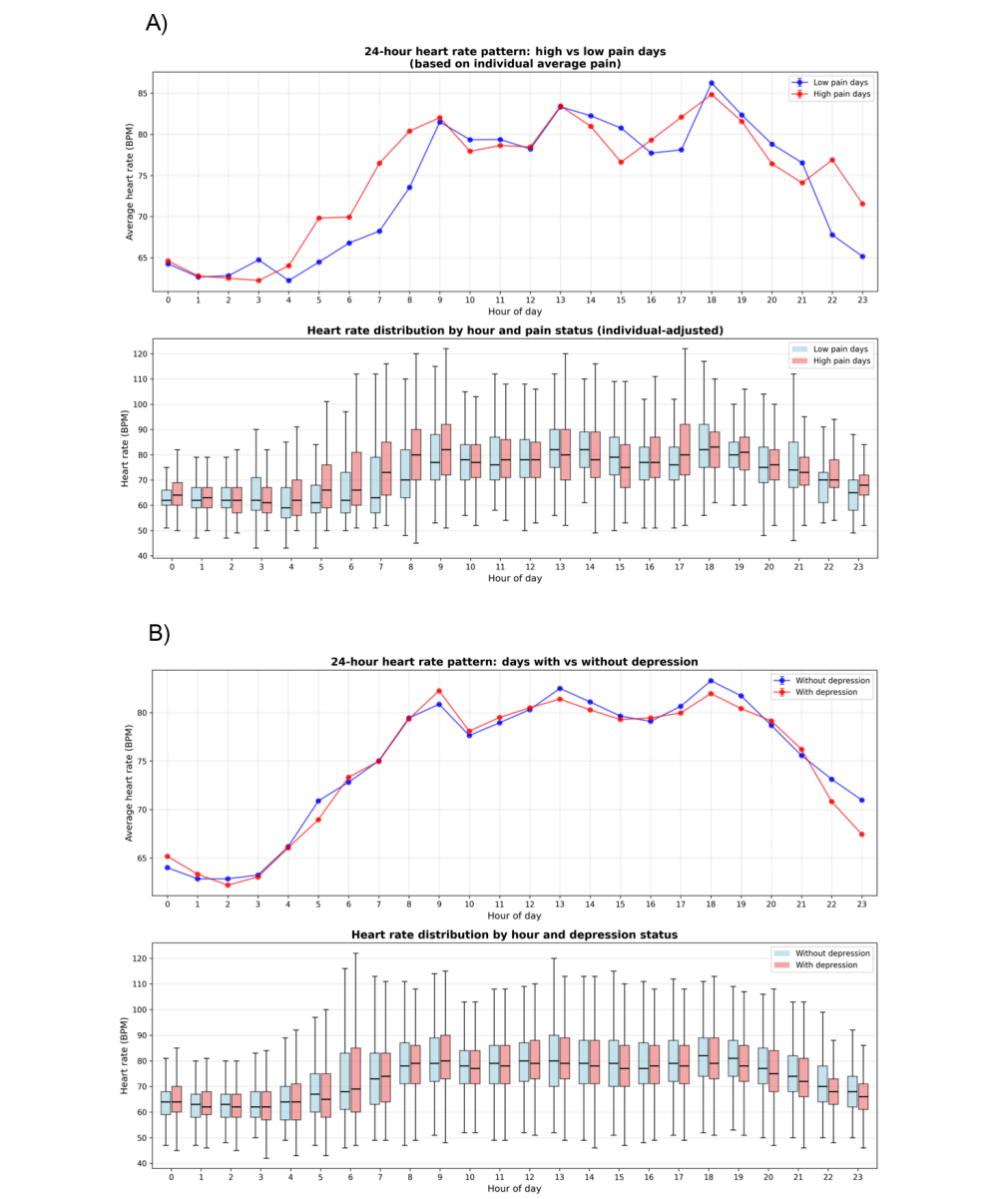
Distinct patterns associated with pain in older adults emerging in 24-hour heart rate profiles. (A) Heart rate patterns on days with higher (red) versus lower (blue) pain relative to individual average demonstrate elevated rates during early morning (5-8 AM) and late evening (10-11 PM) hours, (B) heart rate patterns on days with (red) versus without (blue) self-reported depressive symptoms show minimal variation across the day. Data represent mean (standard error of the mean) from 35 participants over 1318 observation days. Top panels show hourly averages with error bars; bottom panels display distributions as box plots with median, IQR, and outliers. BPM: beats per minute.

We analyzed 1318 days of continuous monitoring data from 35 intervention participants (mean 37.7, SD 8.8 days per participant). Distinct patterns associated with daily pain states were revealed by 24-hour heart rate profiles (Figure 4). Participants experiencing higher-than-usual pain demonstrated elevated heart rates during early morning (5-8 AM) and late evening (10-11 PM) hours compared to their low-pain days (Figure 4A). In contrast, days with and without depressive symptoms showed similar heart rate patterns throughout the day (Figure 4B).

Correlation analyses revealed complex within-person and between-person associations among digital biomarkers and symptoms. Within-person correlations showed that daily fluctuations in sleep time (*r*=–0.112; *P*=.02) and sleep fragmentation (*r*=–0.114; *P*=.01) significantly correlated with depressive symptoms (Table S1 in Multimedia Appendix 2). Between-person correlations indicated that individuals with higher average daily pain levels had higher baseline WOMAC scores (*r*=0.499; *P*=.003), greater average daily depressive symptoms (*r*=0.475; *P*=.006), and greater baseline depressive symptoms (*r*=0.593; *P*<.001; Table S2 in Multimedia Appendix 2). Higher daily depressive symptoms are also associated with root-mean-square of successive differences (*r*=0.356; *P*=.05), moderate PA (*r*=0.462; *P*=.02), and baseline depressive symptoms (*r*=0.476; *P*=.006).

### Digital Predictors of Daily Pain and Depressive Symptoms

Multilevel modeling identified specific digital biomarkers predicting daily pain and depressive symptoms (Table 3). For pain, HRV (mean of the normal-to-normal intervals) significantly predicted both same-day pain (*β*=–.007, SE=0.003; *P*=.02) and next-day pain (*β*=.007, SE=0.003; *P*=.03). Moderate PA was associated with reduced same-day pain (*β*=–.601, SE=0.298; *P*=.047), while increased long wake episodes during sleep predicted higher next-day pain (*β*=.343, SE=0.151; *P*=.03). For depressive symptoms, participants were more likely to report symptoms on days following shorter sleep duration (odds ratio [OR] 0.998, 95% CI 0.997-1.000; *P*=.03) and lower sleep efficiency (OR 0.988, 95% CI 0.975-1.000; *P*=.05). Higher pain increased the likelihood of same-day depressive symptoms (OR 1.222, 95% CI 1.000-1.494; *P*=.05).

**Table 3:** Digital biomarkers predict daily pain and depressive symptoms in older adults. Multilevel models examine within-person associations between sensor-derived measures and self-reported symptoms over 1318 observation days from 35 participants. All predictors were person mean–centered to re-present deviations from individual baselines. Models adjusted for age, sex, and chronic conditions.

Digital predictors			Same-day VAS^a^ pain			Next-day VAS pain			Same-day depressive symptoms			Next-day depressive symptoms	
			Estimate (SE)		*P* value	Estimate (SE)	*P* value	OR^b^ (95% CI)		*P* value	OR (95% CI)		*P* value
Daily depressive symptoms			0.683 (0.392)		.08	0.585 (0.408)	.16	—^c^		—	—		—
Daily pain			—		—	—	—	1.222 (1.000-1.494)		.05	1.132 (.901-1.423)		.28
**Heart rate variability**													
	HF/LF^d^	–0.165 (1.584)		.92		1.258 (2.039)	.54	0.969 (0.170-5.514)		.97	1.060 (0.129-8.727)		.96
	SDNN^e^	–0.005 (0.006)		.44		–0.007 (0.007)	.27	0.450 (0.141-1.443)		.18	1.002 (0.996-1.008)		.56
	Mean NNI^f^	–0.007 (0.003)		.02		0.007 (0.003)	.03	0.998 (0.996-1.001)		.28	1.000 (0.997-1.003)		.85
	RMSSD^g^	–1.668 (0.989)		.09		1.752 (1.285)	.18	0.450 (0.141-1.442)		.18	0.288 (0.079-1.057)		.06
**Sleep**													
	Total sleep time	0.001 (0.001)		.34		0.000 (0.002)	.80	0.998 (0.997-1.000)		.03	1.001 (0.999-1.002)		.32
	Sleep fragmentation index	0.324 (0.234)		.17		0.508 (0.385)	.19	1.142 (0.815-1.602)		.44	1.317 (0.891-1.948)		.17
	Number of long sleep fragmentations	0.148 (0.117)		.21		0.343 (0.151)	.03	1.019 (0.902-1.153)		.76	1.031 (0.902-1.177)		.66
	Sleep efficiency (%)	–0.005 (0.012)		.65		–0.011 (0.013)	.42	0.988 (0.975-1.000)		.05	0.992 (0.979-1.005)		.20
**Physical activity**													
	Light PA^h^	0.002 (0.002)		.25		–0.003 (0.002)	.09	1.000 (0.998-1.002)		.82	1.002 (0.999-1.004)		.16
	Moderate PA	–0.601 (0.298)		.047		0.264 (0.364)	.47	0.996 (0.730-1.358)		.98	1.315 (0.934-1.851)		.12
	Intense PA	–0.256 (0.307)		.41		0.228 (0.354)	.52	0.852 (0.637-1.139)		.28	1.283 (0.921-1.789)		.14

^a^VAS: visual analog scale.

^b^OR: odds ratio.

^c^Not available.

^d^HF/LF: high frequency/low frequency ratio.

^e^SDNN: SD of the normal-to-normal intervals.

^f^Mean NNI: mean of the normal-to-normal intervals.

^g^RMSSD: root-mean-square of successive differences.

^h^PA: physical activity.

In unadjusted analyses, HRV (mean of the normal-to-normal intervals) was associated with both same-day and next-day pain (*P*<.05), and sleep duration was associated with next-day depressive symptoms (*P*<.05), providing initial support for H1. However, after Bonferroni correction for multiple testing (adjusted α=.005), none of these associations remained statistically significant. PA showed limited associations with mood outcomes across all analyses. Therefore, H1 was not supported.

### Intervention Effects on Health Outcomes

**Figure 5:**
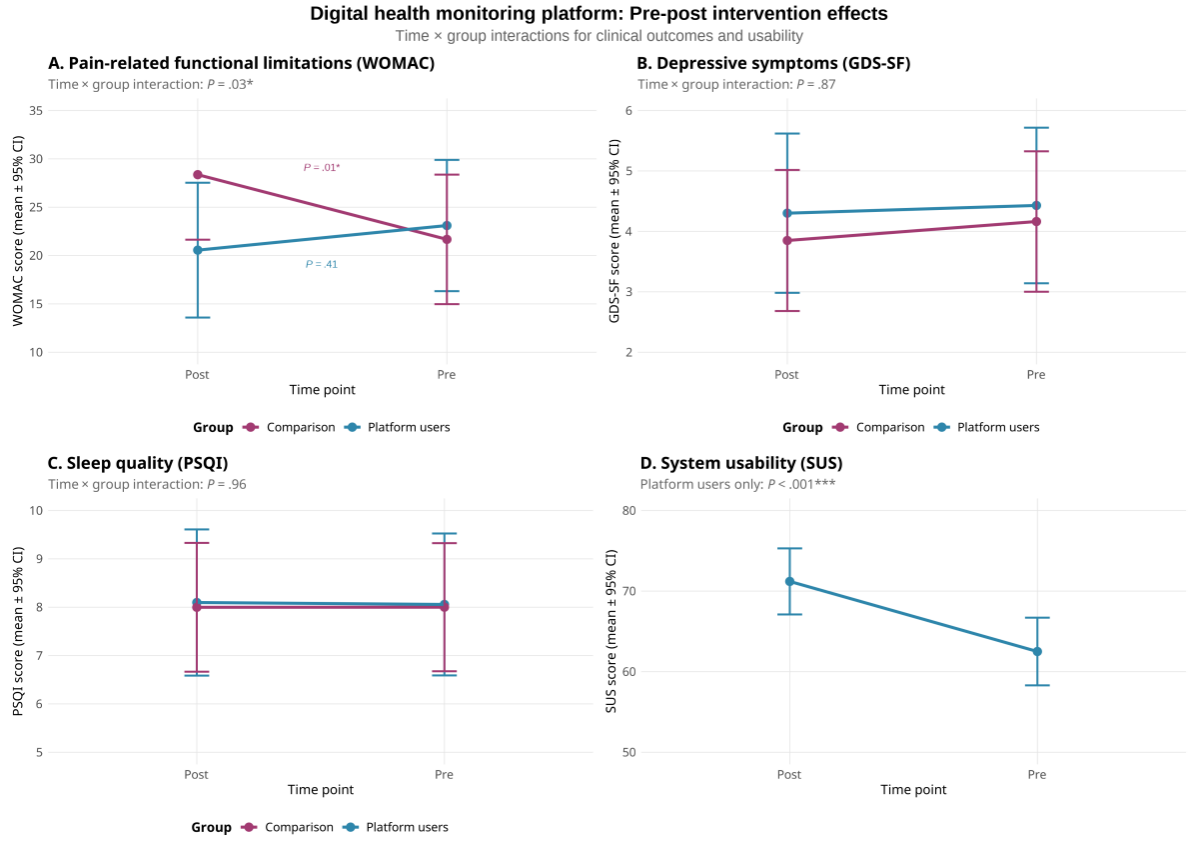
Changes in health outcomes over 6 weeks comparing integrated intervention with usual care. Pre-post intervention changes comparing platform users (blue) with the usual care comparison group (red). (A) Pain-related functional limitations (Western Ontario and McMaster Universities Osteoarthritis Index [WOMAC]) showed significant group × time interaction (F_1,72_=5.26; ^P^=.03), with platform users maintaining function (mean change –2.6, 95% CI –7.8 to 2.6) while the comparison group reported increased functional limitations (mean change +6.7, 95% CI 2.3 to 11.1). (B) Depressive symptoms (Geriatric Depression Scale Short-Form [GDS-SF]), (C) sleep quality (Pittsburgh Sleep Quality Index [PSQI]), and (D) system usability (System Usability Scale [SUS], platform users only) showed no significant changes. Data represent estimated marginal means (95% CI) adjusted for age, sex, and number of chronic conditions. Asterisk (*) indicates a significant interaction effect. Lower scores indicate better outcomes for WOMAC, GDS-SF, and PSQI; higher scores indicate better usability for SUS.

The integrated intervention (digital monitoring plus enhanced caregiver support) yielded differential effects on health outcomes (Figure 5). Intervention participants maintained baseline levels of functional limitations (baseline: mean 23.1, SD 3.4 to post: mean 20.6, SD 3.5; change –2.6, 95% CI –7.8 to 2.6; *P*=.41), while the comparison group reported a significant increase in functional limitations (baseline: mean 21.7, SD 3.4 to post: mean 28.4, SD 3.4; change +6.7, 95% CI 2.3-11.1; *P*=.01). The between-group difference at postintervention was 7.8 (95% CI –16.3 to 0.6; *P*=.07) points, representing a potentially clinically meaningful difference that warrants confirmation in adequately powered trials (Figure 5A).

No significant group differences emerged for depressive symptoms (Figure 5B) or sleep quality (Figure 5C). Both groups maintained stable scores on these measures. System usability among intervention participants showed no significant changes from pre (mean 46.8, SD 20.5) to post (mean 44.9, SD 18.3) intervention (Figure 5D).

These findings partially support H2. Intervention participants demonstrated maintained functional health while the comparison group reported increased functional limitations, supporting our hypothesis for pain-related outcomes. However, the hypothesis was not supported for depressive symptoms or sleep quality, which showed no significant group differences.

### Platform Engagement and Care Coordination

Despite limited baseline digital literacy, platform engagement remained robust throughout the intervention. Daily chatbot survey completion averaged 65% (863/1318), with 73% (962/1318) compliance for heart rate monitoring, 53% (693/1318) for sleep tracking, and 90% (1192/1318) for activity monitoring. Missing data primarily resulted from device charging requirements or forgetting to wear devices rather than technical difficulties.

Table 2 presents caregiver’s app usage at posttest. While maintaining the regular working hours (mean 25, SD 1.3 hours per week), community caregivers in the intervention group demonstrated strong platform engagement, with acceptable usability scores (mean SUS score 70.8, SD 16.7) and frequent daily use (16/23, 69.5% used ≥2-3 times daily). Caregivers actively used multiple functions (mean 6.0, SD 3.9), most commonly accessing voice recordings (19/23, 82.6%), step monitoring (14/23, 60.9%), and stress level tracking (13/23, 56.5%), indicating comprehensive adoption of the platform’s digital monitoring features.

The platform appeared to enhance existing care services through 37 health decline–triggered interventions (Table S3 in Multimedia Appendix 1 for case reports and Figure S1 in Multimedia Appendix 1 for platform alert screenshots). When the platform detected concerning patterns (multiple red indicators for ≥2 consecutive days), caregivers conducted targeted interventions addressing: severe postprocedure pain (n=3), dizziness requiring medical attention (n=5), medication nonadherence (n=2), infections requiring hospitalization (n=1), and physical inactivity (n=26). One critical intervention identified an infected abscess requiring immediate surgical drainage, potentially preventing sepsis. All 25 community caregivers completed the study and actively responded to health alerts, demonstrating successful integration of digital monitoring with in-person care delivery.

Regarding safety, no serious adverse events were directly attributable to the platform. While 4 intervention participants withdrew citing burden and 3 participants discontinued due to unrelated illness or relocation, the 37 health decline–triggered interventions represent early detection rather than platform-induced harms.

H3 was supported. All 25 community caregivers completed the study and successfully integrated platform alerts into their workflows, conducting 37 health decline–triggered interventions. Poststudy surveys confirmed that caregivers did not exceed their contracted 25-hour work week, demonstrating that platform integration was achieved without additional work hours or service costs beyond the standardized care protocol.

## Discussion

### Principal Findings

This feasibility study provides preliminary evidence that integrating digital health monitoring with existing community care infrastructure may help bridge the digital divide for low-income older adults. The primary observation—that participants receiving the integrated intervention maintained functional health while the comparison group reported increased functional limitations—is consistent with potential benefits of the integrated digital-human care model, though the nonrandomized, quasi-experimental design precludes causal inference regarding which intervention components contributed to this differential pattern. The 37 health decline–triggered interventions by digital monitoring illustrate how technology may complement existing, rather than replace, human care relationships. However, it is important to note that the intervention group received caregiver interventions triggered by digital monitoring, while controls received standard care only. This fundamental confounding prevents isolation of digital platform effects from enhanced human contact. Our findings support the feasibility and preliminary effectiveness of digital-human integration but cannot establish independent clinical efficacy of the digital platform itself.

Our digital phenotyping results provide novel insights into pain and mood fluctuations among community-dwelling older adults. The distinct 24-hour heart rate patterns associated with pain—elevated rates during early morning and late evening—may reflect circadian variations in pain perception, previously observed only in controlled laboratory settings [59,60] but now demonstrated in older adults’ daily living conditions. The association between HRV and both same-day and next-day pain extends previous cross-sectional findings of lower HRV in chronic pain patients [61,62], providing the first evidence that within-person HRV fluctuations may predict daily pain variations in older adults, suggesting autonomic dysregulation as an early indicator of pain exacerbation. Similarly, the relationship between sleep quality metrics and depressive symptoms aligns with established bidirectional links between sleep and mood in geriatric populations [63,64].

Regarding our hypotheses, results provided support for H2 and H3, but H1 was not supported after statistical correction. The digital phenotyping hypothesis (H1) was not supported: while HRV and sleep metrics showed initial associations with pain and depressive symptoms in unadjusted analyses, these relationships did not survive Bonferroni correction for multiple testing (adjusted α=.005). The effectiveness hypothesis (H2) received partial preliminary support: a differential pattern in functional health was observed, favoring the intervention group, but depression and sleep outcomes showed no group differences, possibly due to the short intervention duration and the confounding effects of enhanced caregiver support. The feasibility hypothesis (H3) was fully supported: caregivers successfully integrated digital monitoring into existing workflows without additional time burden, as confirmed by poststudy surveys verifying adherence to the standardized 25-hour weekly schedule. This finding is relevant for potential scalability, raising the hypothesis that integrated interventions may enhance care quality through better timing of interventions rather than requiring additional caregiver burden. This hypothesis requires confirmation through randomized designs.

### Comparison With Prior Work

Previous digital health interventions for older adults have reported mixed results, with high attrition rates often limiting effectiveness [65,66]. A recent systematic review found that internet-delivered interventions for geriatric depression achieved modest effects but struggled with engagement, particularly among those with lower digital literacy [67-69]. Our approach differs fundamentally by not requiring direct technology use by older adults themselves. This intermediary model extends traditional community health worker programs—proven effective in resource-limited settings [70-73]—by integrating digital monitoring tools with trained lay caregivers who facilitate early detection and timely intervention for mental and physical health concerns among vulnerable populations.

Our findings extend the digital phenotyping literature, which has predominantly focused on younger populations with sufficient digital literacy [4,16,74-76]. While previous studies have shown associations between PA and mood in controlled settings [77-80], our real-world data revealed more complex and weaker patterns that did not reach statistical significance. The observed trend toward a protective effect of moderate—but not intense—PA on pain aligns with recommendations for gradual, individualized exercise in older adults [81,82], though further research is needed to establish whether digital monitoring could help identify optimal activity ranges for personalized pain management [5].

### Implications for Digital Health Equity

This study offers a preliminary model that, if validated through randomized trials, may be scalable for addressing digital health inequities. Rather than attempting to overcome multiple barriers simultaneously—teaching digital skills, providing devices, ensuring connectivity—our approach leverages 1 existing asset: regular contact with community caregivers. This transforms the digital literacy requirement from end users to intermediaries who typically possess better digital skills and can translate complex health data into actionable care for service-receiving older adults.

The current implementation of the developed platform in South Korea, where public community care services are well-established, suggests potential adaptability to other contexts with similar infrastructure. Many countries have comparable programs—community health worker programs operating globally [83,84]—that offer ready infrastructure for deploying digital health technologies to support aging populations.

### Clinical and Policy Implications

Our findings suggest that digital health equity may require more than providing devices or training programs. Successful implementation depends on integrating technology within existing care relationships and workflows [23,70]. For clinicians, this means recognizing that digital health tools may be most effective when delivered through care teams rather than directly to patients. For policymakers, investing in community care infrastructure may paradoxically be essential for realizing the benefits of digital health investments.

The cost-effectiveness and scalability of this approach warrant future investigation. Implementation costs include (1) 1-time expenses for smartwatches (approximately US $200 per participant), smartphone app development, and server infrastructure; (2) recurring costs for device replacement, data storage, and technical support; and (3) caregiver time and wages for platform monitoring and responsive interventions. Notably, this study confirmed that these costs may be partially offset by integration with existing community care workflows—caregivers already conduct scheduled contacts, and platform monitoring required approximately 10-15 additional minutes daily per 10 assigned recipients for reviewing dashboard alerts. The critical economic question is whether early detection prevents costly downstream events. In our 6-week trial, the 37 triggered interventions included 1 case of infected abscess identified before sepsis development, suggesting substantial potential for preventing emergency department visits and hospitalizations that would far exceed platform costs. Formal cost-effectiveness analysis comparing platform-enhanced care versus usual care, incorporating quality-adjusted life years and health care use outcomes, is essential before recommending widespread implementation [85].

The below-threshold system usability scores (mean SUS score 46, SD 21.5 versus the cutoff score of 68 for acceptable usability) warrant consideration. These low scores likely reflect our target population’s limited baseline digital literacy rather than platform-specific usability failures, as participants reported difficulty identifying any digital technology for reference during baseline assessment and no available internet connections at home. Nevertheless, suboptimal usability may have attenuated intervention effects by limiting engagement depth or creating frustration that offset potential benefits. In contrast, caregivers demonstrated acceptable usability scores and high engagement, with 69.5% (16/23) using the platform 2-3 times daily. Future iterations should prioritize simplified interfaces, voice-first interaction modalities, and extended onboarding support for older adults. Additionally, the low SUS scores suggest that the intermediary model—where caregivers translate platform outputs rather than expecting direct user proficiency—may be essential rather than merely convenient for this population.

### Limitations

Several limitations warrant consideration. First, the quasi-experimental, nonrandomized design with unequal intervention exposure introduces fundamental confounding that limits causal inference. The intervention group received platform-triggered caregiver interventions, while the control group received standard scheduled care. This design makes it impossible to isolate the effect of the digital platform from the effect of possibly increased human contact, limiting conclusions to the feasibility and preliminary effectiveness of the integrated care model rather than independent platform efficacy. Randomization was not feasible in part because the multiphase research program spanned the COVID-19 pandemic and postpandemic periods (2021-2023), during which protecting vulnerable participants’ safety and informed decision-making were prioritized. Voluntary participation through prior consultation with trusted caregivers was a prerequisite for enrollment, consistent with the participatory development process. This necessitated a matched comparison design based on caregiver and participant willingness rather than random assignment. Although the current living lab approach provides ecological validity for understanding real-world implementation within existing care systems, it was not feasible to match care services for the structured control group or require the provision of detailed reports of caregiving service types and the amount of time for each participant in both the intervention and control groups.

The nonrandomized design may have introduced additional potential selection bias, though groups were well-matched on measured characteristics. Particularly, intervention group caregivers volunteered to adopt the platform, potentially selecting for caregivers with greater technological comfort, higher motivation, or stronger relationships with their care recipients—factors that could independently affect health outcomes. The open-label nature was also unavoidable given the intervention’s visibility to both participants and caregivers. Notably, both groups were drawn from older adults with established caregiver relationships who consented to face-to-face research assessments during the postpandemic period—a decision that itself reflects a degree of trust and social engagement uncommon in this population. The comparison group thus represents a relatively engaged subgroup rather than a passive usual-care population, which may partially mitigate concerns about selection bias favoring the intervention group.

Additionally, our multicomponent intervention does not allow experimental isolation of active mechanisms, and findings should not be interpreted as identifying which specific components drove the observed differential patterns. The platform integrated (1) continuous physiological monitoring via smartwatches, (2) daily chatbot surveys for symptom reporting, (3) personalized traffic-light feedback to users, (4) real-time dashboard alerts to caregivers, and (5) protocol-driven caregiver responses to health decline signals. Any or all components may have contributed to observed effects, individually or synergistically. For example, improvements could result from increased self-monitoring awareness, the supportive effect of daily chatbot interaction, enhanced caregiver responsiveness, or the earlier timing of follow-up enabled by digital alerts, or from complex interactions among these elements. Future research should use factorial designs or sequential multiple assignment randomized trials to systematically isolate component contributions and identify optimal intervention configurations for different patient subgroups.

While the multicomponent design precludes experimental isolation of active mechanisms, several observations generate testable hypotheses for future studies designed to isolate component effects. The 37 caregiver-initiated interventions were triggered by digital alerts rather than scheduled contacts, suggesting that the platform’s primary mechanistic contribution may lie in the timing of care delivery—enabling earlier detection and response to health deterioration—rather than in the volume of caregiver contact. Caregivers maintained their standard 25-hour weekly schedule, further suggesting that differential outcomes may reflect improved allocation of existing care resources rather than increased service intensity. Additionally, the contrast between low older adult usability scores (mean SUS score 46, SD 21.5) and acceptable caregiver usability scores (mean SUS score 70.8, SD 16.7) suggests that intervention effects likely operated primarily through the caregiver-mediated pathway rather than through direct older adult engagement with the platform. These observations point toward caregiver-mediated early detection as a candidate mechanism, but this hypothesis requires formal testing through factorial designs that systematically vary platform components.

In addition, the 6-week intervention period represents a significant constraint. While adequate for demonstrating feasibility and detecting changes in functional limitations, this duration is insufficient for assessing depression outcomes, which meta-analytic evidence suggests require longer intervention periods [86]. Furthermore, observed effects may partly reflect novelty or Hawthorne effects that could diminish over time as participants habituate to monitoring. Long-term studies are needed to determine whether functional benefits persist, whether initially null effects on depression emerge with extended exposure, and whether engagement levels remain sustainable beyond the initial phase.

Also, missing data rates for some sensors (particularly sleep monitoring, 693/1318, 52.6%) may have limited our ability to detect associations, and nonrandom missingness cannot be excluded. Our sample of urban-dwelling Korean older adults limits generalizability to rural populations or other cultural contexts. The marginal significance of several findings after multiple testing corrections suggests the need for larger studies to confirm these associations.

Finally, our daily depression assessment—2 randomly selected binary PHQ-9 items—has limited sensitivity to mood fluctuations compared to full validated scales or ecological momentary assessment approaches with continuous response scales. This measurement constraint may have reduced our ability to detect subtle associations between digital biomarkers and mood states, potentially contributing to the null findings for depression outcomes. Future studies should use more sensitive daily mood measures, such as VASs or brief validated instruments designed for repeated administration.

### Conclusions

This feasibility study provides preliminary evidence suggesting that integrating digital monitoring within existing community infrastructure may advance digital health support to low-income older adult populations. By positioning formal caregivers as technology intermediaries, vulnerable older adults were able to participate in continuous multimodal health monitoring while addressing digital literacy barriers. This approach demonstrated feasibility and raised the question of whether digital health equity may be better addressed by reimagining how, not just what, technologies are delivered within the existing community care infrastructure. Future research should use randomized controlled designs with appropriate control conditions to establish causal relationships and isolate the specific contributions of digital monitoring, enhanced feedback, and caregiver responsiveness to the observed differential patterns. Such studies will be essential for determining optimal intervention and implementation strategies across diverse settings and evaluating the long-term sustainability of this integrated care model.

## Appendix

Multimedia Appendix 1 Higher resolution version of Figure 2.

Multimedia Appendix 2 Within- and between-person correlations of daily health measures; follow-up records after detected deterioration; and examples of caregiver app health decline reports.

## Abbreviations

GDS-SF: Geriatric Depression Scale Short-Form
H1: hypothesis 1
H2: hypothesis 2
H3: hypothesis 3
HRV: heart rate variability
OR: odds ratio
PA: physical activity
PHQ-9: Patient Health Questionnaire-9
PSQI: Pittsburgh Sleep Quality Index
SUS: System Usability Scale
VAS: visual analog scale
WOMAC: Western Ontario and McMaster Universities Osteoarthritis Index
