# Supplementary figures and images for: Digital-Human Public Community Care Integration for Chronic Pain in Low-Income Older Adults in a 6-Week Living Lab Setting: Quasi-Experimental Feasibility Study
Source: JMIR Aging. 2026 Apr 20;9:e85611. doi: 10.2196/85611 (PMC13139835; doi:10.2196/85611)

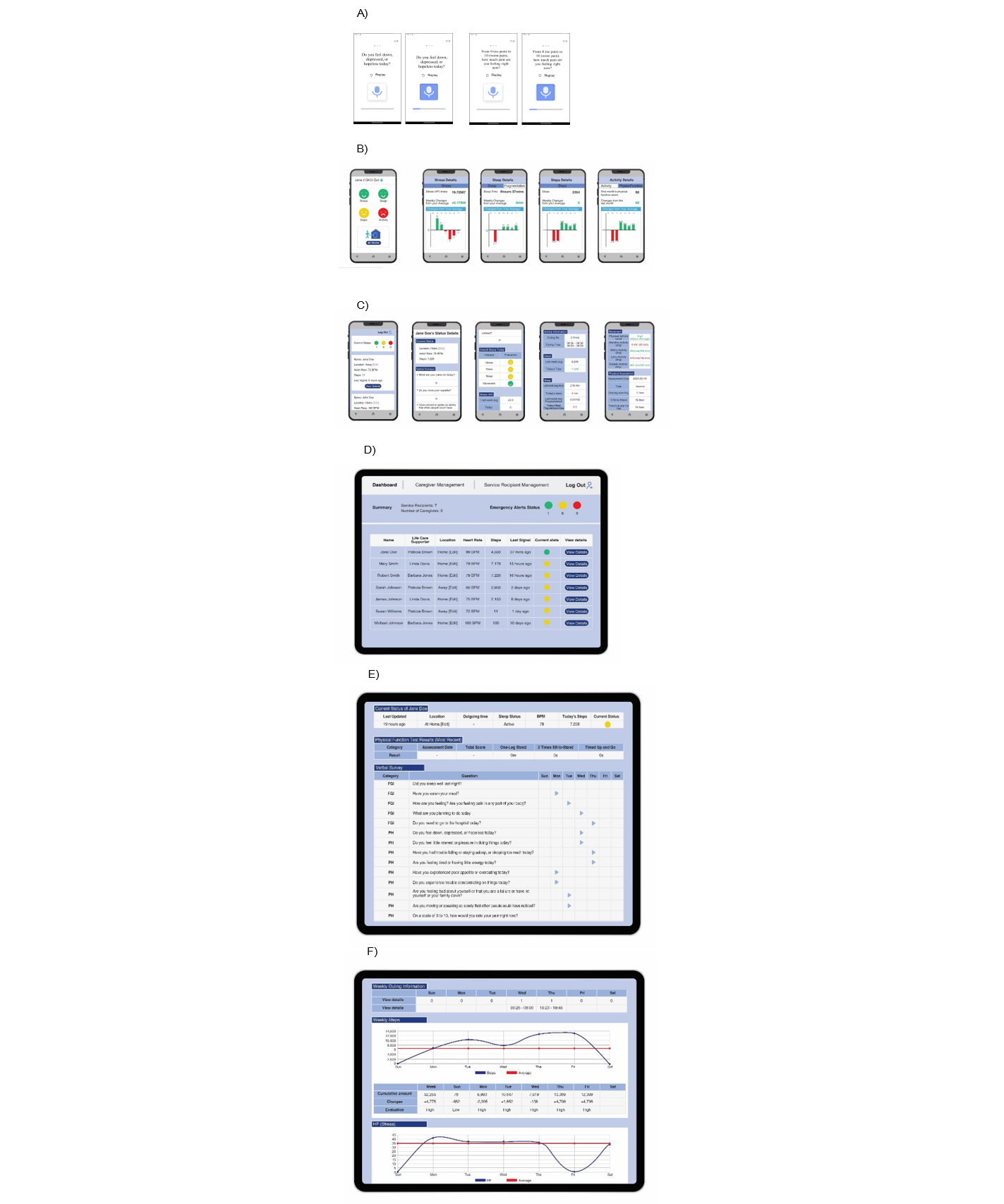

Supplement: Multimedia Appendix 1 [file aging_v9i1e85611_app1.png]
