# Supplementary material for: Digital-Human Public Community Care Integration for Chronic Pain in Low-Income Older Adults in a 6-Week Living Lab Setting: Quasi-Experimental Feasibility Study
Source: JMIR Aging. 2026 Apr 20;9:e85611. doi: 10.2196/85611 (PMC13139835; doi:10.2196/85611)
Supplement: Multimedia Appendix 2 [file aging_v9i1e85611_app2.docx]

**Supplemental materials**

**Table S1. Within-person correlations among daily measures of pain, depressive symptoms, heart rate variability, sleep, and physical activity indicators.**

|  | **1** | **2** | **3** | **4** | **5** | **6** | **7** | **8** | **9** | **10** | **11** | **12** |
| --- | --- | --- | --- | --- | --- | --- | --- | --- | --- | --- | --- | --- |
| **1. Daily pain** | - |  |  |  |  |  |  |  |  |  |  |  |
| **2. Daily depression** | 0.054 | - |  |  |  |  |  |  |  |  |  |  |
| **3. HF/LF** | -0.031 | 0.047 | - |  |  |  |  |  |  |  |  |  |
| **4. SDNN** | 0.033 | -0.052 | **0.325^***^** | - |  |  |  |  |  |  |  |  |
| **5. Mean NNI** | 0.008 | -0.055 | **0.450^***^** | **0.135^***^** | - |  |  |  |  |  |  |  |
| **6. RMSSD** | 0.022 | 0.009 | -0.105* | 0.055 | **0.147^***^** | - |  |  |  |  |  |  |
| **7. Sleep time** | 0.117 | **-0.112^*^** | -0.039 | **0.118^**^** | **0.240^***^** | **0.220^***^** | - |  |  |  |  |  |
| **8. Sleep fragmentation index** | 0.032 | **-0.114^*^** | -0.023 | 0.006 | -0.023 | -0.019 | **0.223^***^** | - |  |  |  |  |
| **9. No. of long sleep fragmentation** | -0.08 | 0.026 | **-0.253^***^** | **-0.332^***^** | **-0.687^***^** | **-0.687^***^** | **-0.122^**^** | 0.065 | - |  |  |  |
| **10. Sleep efficiency** | 0.062 | -0.030 | -0.066 | **0.142^***^** | **0.257^***^** | 0.085 | **0.270^***^** | **-0.566^***^** | **-0.365^***^** | - |  |  |
| **11. Light PA** | 0.082 | -0.020 | **0.172^***^** | **0.239^***^** | **0.129^***^** | 0.062 | 0.043 | **0.329^***^** | **-0.270^***^** | **-0.168^***^** | - |  |
| **12. Moderate PA** | 0.151 | -0.018 | -0.048 | **0.150^**^** | **0.149^**^** | **0.114^*^** | **0.130*** | **-0.298^***^** | **-0.236^***^** | **0.233^***^** | **0.137^**^** | - |
| **13. Intense PA** | -0.019 | 0.008 | -0.010 | -0.049 | **-0.309^***^** | -0.126 | **0.151*** | 0.055 | **0.502^***^** | **-0.303^***^** | **-0.162^**^** | -0.068 |

**Note.** *** p ≤ 0.001, ** p ≤ 0.01, * p ≤ 0.05; HF/LF: High Frequency/Low Frequency ratio, SDNN: Standard Deviation of the N-N intervals, Mean NNI: Mean of the Normal-to-Normal Intervals, RMSSD: Root Mean Square of Successive Differences, PA: Physical Activity

**Table S2. Between-person correlations among daily measures of pain, depressive symptoms, heart rate variability, sleep, and physical activity indicators and pretest measures of pain, depressive symptoms, sleep quality, and social isolation.**

|  | **1** | **2** | **3** | **4** | **5** | **6** | **7** | **8** | **9** | **10** | **11** | **12** | **13** | **14** | **15** | **16** |
| --- | --- | --- | --- | --- | --- | --- | --- | --- | --- | --- | --- | --- | --- | --- | --- | --- |
| **1. Daily pain** | - |  |  |  |  |  |  |  |  |  |  |  |  |  |  |  |
| **2. Daily depression** | **0.475^**^** | - |  |  |  |  |  |  |  |  |  |  |  |  |  |  |
| **3. HF/LF** | -0.252 | 0.014 | - |  |  |  |  |  |  |  |  |  |  |  |  |  |
| **4. SDNN** | 0.042 | 0.009 | **0.621^***^** | - |  |  |  |  |  |  |  |  |  |  |  |  |
| **5. Mean NNI** | 0.086 | -0.130 | **0.525^**^** | **0.854^***^** | - |  |  |  |  |  |  |  |  |  |  |  |
| **6. RMSSD** | 0.128 | **0.356^*^** | -0.009 | 0.017 | -0.132 | - |  |  |  |  |  |  |  |  |  |  |
| **7. Sleep time** | 0.283 | -0.140 | -0.011 | 0.071 | 0.260 | -0.287 | - |  |  |  |  |  |  |  |  |  |
| **8. Sleep fragmentation** | 0.216 | 0.183 | 0.284 | -0.044 | -0.057 | 0.345 | 0.254 | - |  |  |  |  |  |  |  |  |
| **9. Long sleep fragmentation** | -0.136 | 0.149 | 0.248 | -0.04383 | 0.008 | -0.004 | -0.171 | -0.119 | - |  |  |  |  |  |  |  |
| **10. SE** | -0.033 | 0.122 | -0.351 | -0.336 | **-0.450^**^** | **0.598^***^** | -0.267 | 0.023 | -0.034 | - |  |  |  |  |  |  |
| **11. Light PA** | 0.154 | 0.045 | 0.364* | **0.603^***^** | **0.576^***^** | -0.159 | 0.318 | 0.194 | -0.041 | **-0.586^***^** | - |  |  |  |  |  |
| **12. Moderate PA** | 0.251 | **0.462^*^** | -0.157 | -0.258 | -0.317 | **0.663^***^** | -0.177 | 0.105 | -0.102 | **0.469^*^** | -0.374 | - |  |  |  |  |
| **13. Intense PA** | 0.107 | -0.063 | **-0.494^*^** | -0.061 | 0.025 | **-0.596^*^** | **0.476^*^** | -0.386 | -0.187 | -0.272 | 0.109 | **-0.575^*^** | - |  |  |  |
| **14. Pretest WOMAC** | **0.499^**^** | 0.104 | -0.152 | 0.064 | 0.250 | 0.021 | 0.010 | -0.044 | -0.132 | -0.213 | 0.115 | 0.220 | -0.295 | - |  |  |
| **15. Pretest GDS** | **0.593^***^** | **0.476^**^** | -0.220 | -0.035 | 0.161 | 0.156 | 0.232 | 0.024 | -0.064 | 0.095 | -0.100 | 0.231 | 0.240 | 0.286 | - |  |
| **16. Pretest PSQI** | 0.297 | -0.045 | 0.111 | 0.339 | **0.407^*^** | -0.241 | 0.016 | 0.044 | 0.099 | **-0.448^**^** | **0.361^*^** | **-0.459^*^** | -0.065 | **0.455^**^** | 0.187 | - |
| **17 Pretest LSI** | -0.198 | 0.046 | **0.384^*^** | **0.380^*^** | **0.392^*^** | 0.171 | -0.297 | -0.195 | 0.302 | 0.118 | -0.019 | -0.210 | -0.135 | -0.047 | 0.044 | **0.379^*^** |

**Note.** *** p ≤ 0.001, ** p ≤ 0.01, * p ≤ 0.05; HF/LF: High Frequency/Low Frequency ratio, SDNN: Standard Deviation of the N-N intervals, Mean NNI: Mean of the Normal-to-Normal Intervals, RMSSD: Root Mean Square of Successive Differences, PA: Physical Activity

WOMAC: Western Ontario and McMasters Universities Osteoarthritis Index, GDS: Geriatric Depression Scale, PSQI: Pittsburg Sleep Quality Index.

**Table S3. Records of the in-person follow-ups after detecting health deterioration in daily monitoring.**

| **No.** | **Date** | **Detected Health Decline** | **In-Person Follow-Up Outcome** |
| --- | --- | --- | --- |
| 1 | 23. 10. 04 | - Participant A: Continuous signs of reduced activity and poor sleep detected. | - A community caregiver discovered the older adults in severe post-tooth extraction pain (10/10). Sleep began to recover; walking and sleep indicators changed from warning to normal the next day. |
| 2 | 23. 10. 04 | - Participant B reported experiencing depressive symptoms for over a week during a verbal interview, although their tone and speech pace remained strong. | - Through the community caregiver’s visit, it was confirmed that the elder was engaging in enjoyable daily activities as usual, such as participating in church gatherings. |
| 3 | 23. 10. 05 | - Participant C: In a verbal response, the elder reported having a painful area and described the pain as severe. They rated the pain as 5 out of 10. | - The next day, a community caregiver conducted an in-person follow-up. The older adult reported numbness in the leg and a swollen lump on the buttock. The caregiver accompanied the older adult to the hospital, where bloody pus was drained and a biopsy was performed to check for malignancy. The case was escalated to intensive care through regular in-person support. |
| 4 | 23. 10. 05 | - Participant D: The elder reported a depressed mood and a pain level of 7 out of 10. Worsening sleep patterns were identified through monitoring. | - According to the community care worker’s visit, the older adult had been experiencing nausea and dizziness since the Thanksgiving holiday. Although their younger daughter invited the older adult for a meal, the older adult was unable to go. However, after visiting an internal medicine clinic, both their condition and nausea significantly improved. |
| 5 | 23. 10. 06 | - Participant B: In a verbal response, the elder spoke with a hoarse voice, reported a sore throat and fatigue, and rated their pain as 7 out of 10. A significant decrease in step count was observed a day before, along with severely fragmented sleep. | - According to the community caregiver's follow-up, the elder had abruptly stopped taking sleep medication. The older adult exhibited signs of anxiety, expressing fear that someone might enter through an open window. However, the older adult felt better when interacting with others. The caregiver provided reassurance and promised continued monitoring and prompt support. |
| 6 | 23. 10. 11 | - Participant C: Both step count and sleep indicators showed critical alerts. | - This was a recurrence following a previous episode of health deterioration. A follow-up by the community care service confirmed that the older adult's condition had improved sufficiently to allow a return to work. |
| 7 | 23. 10. 11 | - Participant E: Critical health warnings were detected across all domains: stress levels, steps, sleep, and overall movement. | - The community caregiver confirmed that the older adult had been unwell over the holidays but is now recovering. |
| 8 | 23. 10. 12 | - Participant D: While a decline in physical activity was detected, sleep and stress indicators remained within normal ranges. | - According to the caregiver’s follow-up, the older adult recovered after getting sufficient sleep the previous night and planned to go for a walk in the afternoon. |
| 9 | 23. 10. 12 | - Participant A: Worsening sleep and stress indicators were detected. | - Caregiver follow-up confirmed that the older adult was in stable condition. |
| 10 | 23. 10. 13 | - Participant F: Signs of increased stress were detected. | - As a result of the follow-up service, it was confirmed that the older adult has early-stage dementia and that the day was her dialysis day, which may have contributed to the elevated stress; however, her condition was consistent with their usual state. |
| 11 | 23. 10. 13 | - Participant B, C, and D: Signs of decreased physical activity were detected. | - The assigned community caregiver encouraged physical activity, such as walking, through their service. |
| 12 | 23. 10. 13 | - Participant C: Signs of decreased physical activity were detected. | - The assigned community caregiver encouraged physical activity, such as walking, through their service. |
| 13 | 23. 10. 13 | - Participant D: Signs of decreased physical activity were detected. | - The assigned community caregiver called to encourage physical activity, such as walking, through their service. |
| 14 | 23. 10. 16 | - Participant D: Signs of decreased physical activity were detected again. | - The assigned community caregiver visited to in-person check-up and encouraged physical activity despite lack of motivations. |
| 15 | 23. 10. 17 | - Participant B, C, D, and H: Signs of decreased physical activity were detected. | - The assigned community caregiver encouraged physical activity, such as walking, through their service. An increase in activity levels was observed the following day. |
| 13 | 23. 10. 19 | - Participant D: Signs of increased stress were detected. | - The follow-up service confirmed that the older adult was in her usual condition. |
| 14 | 23. 10. 20 | - Participant D: Signs of increased stress and decreased physical activity were detected. | - As a result of the follow-up service, it was confirmed that the older adult was in her usual condition, but the caregiver encouraged her to engage in physical activity. |
| 15 | 23. 10. 20 | - Participant B: Signs of increased stress and decreased step count were detected. | - As a result of the follow-up service, it was confirmed that the older adult was in their usual condition, but they were encouraged to engage in physical activity. |
| 16 | 23. 10. 20 | - Participant H: Signs of decreased step count were detected. | - It was confirmed that the older adult was in their usual condition, but they were encouraged to engage in physical activity. |
| 17 | 23. 10. 20 | - Participant E: Signs of decreased step count were detected. | - The caregiver visited the older adult’s home and encouraged her to engage in physical activity. |
| 18 | 23. 10. 20 | - Participant F: Signs of increased stress were detected. | - As a result of the follow-up service, it was confirmed that the older adult was in their usual condition. |
| 19 | 23. 10. 23 | - Participant D: Signs of increased stress and decreased step count were detected. | - According to the community caregiver’s report, the older adult was in her usual condition; the caregiver encouraged physical activity. |
| 20 | 23. 10. 23 | - Participant I: Signs of decreased step count were detected. | - After the community caregiver’s visit, the older adult agreed to engage in physical activity. |
| 21 | 23. 10. 23 | - Participant J: Signs of increased stress and decreased step count were detected. | - Based on the community caregiver’s report, the older adult’s health status was stable. |
| 22 | 23. 10. 24 | - **Participant D**: Signs of increased stress and decreased step count were detected. | - According to the community caregiver’s report, Participant D was in fine health and agreed to engage in physical activity. |
| 23 | 23. 10. 24 | - **Participant I**: Signs of decreased step count were detected. | - According to the community caregiver’s report, Participant I agreed to engage in physical activity in the afternoon. |
| 24 | 23. 10. 24 | - **Participant B**: Signs of decreased step count were detected. | - According to the community caregiver’s check, Participant B agreed to engage in physical activity in the afternoon. |
| 25 | 23. 10. 24 | - **Participant H**: Signs of decreased step count were detected. | - The community caregiver encouraged the participant to engage in physical activity. |
| 26 | 23. 10. 25 | - **Participant D**: Signs of poor sleep were detected. | - As a result of the follow-up, it was confirmed that the participant had experienced a recurrence of dizziness since the previous evening, had visited the hospital, and was currently resting. |
| 27 | 23. 10. 25 | - **Participant B**: Signs of decreased step count were detected. | - The community caregiver encouraged the participant to continue engaging in regular physical activity. |
| 28 | 23. 10. 25 | - **Participant J**: Signs of decreased step count and poor sleep were detected. | - As a result of the follow-up service, it was confirmed that the participant had not slept well the previous night but had no health issues. The participant stated they would engage in physical activity in the afternoon. |
| 29 | 23. 10. 25 | - **Participant I**: Signs of decreased step count were detected. | - The community caregiver encouraged the participant to continue engaging in regular physical activity. |
| 30 | 23. 10. 25 | - **Participant B**: Signs of decreased step count were detected. | - After checking in on the participant, the community caregiver encouraged them to continue engaging in regular physical activity. |
| 31 | 23. 10. 27 | - **Participant D**: Signs of decreased step count were detected. | - As a result of the follow-up service, dizziness had significantly improved, and the participant agreed to begin engaging in physical activity gradually once their condition further improves. |
| 32 | 23. 10. 27 | - **Participant H**: Signs of decreased physical activity were detected. | - The community caregiver encouraged the participant to engage in physical activity. |
| 33 | 23. 10. 27 | - **Participant F**: Signs of increased stress were detected. | - Through the follow-up service, it was confirmed that the participant was safe, in good spirits, and in a condition similar to their usual state. |
| 34 | 23. 10. 27 | - **Participant B**: Signs of decreased physical activity were detected. | - The community caregiver encouraged the participant to engage in physical activity. |
| 35 | 23. 10. 31 | - **Participant D**: Signs of decreased step count were detected. | - As a result of the follow-up service, dizziness had improved, and the participant agreed to engage in physical activity in the afternoon. |
| 36 | 23. 10. 31 | - **Participant F**: Signs of increased stress were detected. | - **According to the community caregiver’s report, the participant was in a condition similar to their usual state.** |
| 37 | 23. 10. 31 | - **Participant B**: Signs of decreased step count were detected. | - According to the community caregiver’s report, the older adult received her phone call and said to engage in physical activity. |

**Figure S1.** Examples of detected health decline reports on the caregiver’s app

| 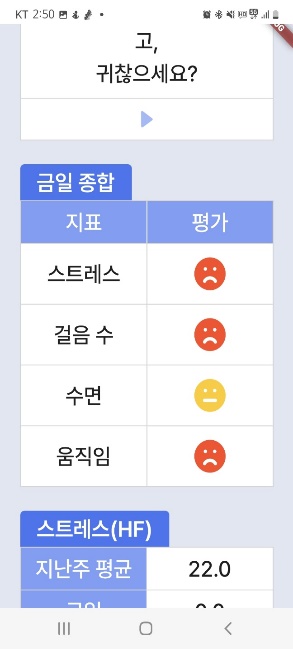 | 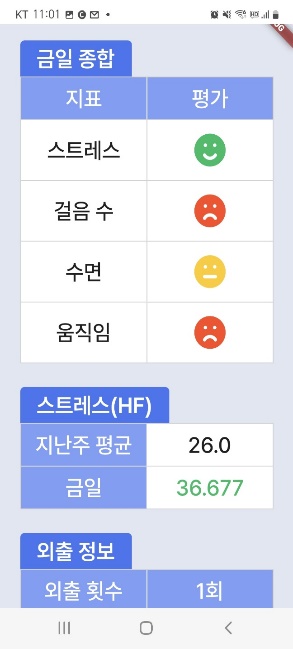 | 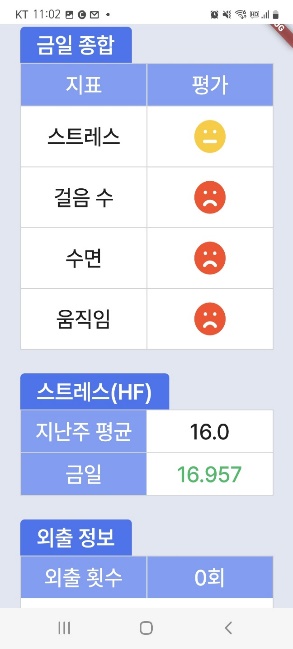 | 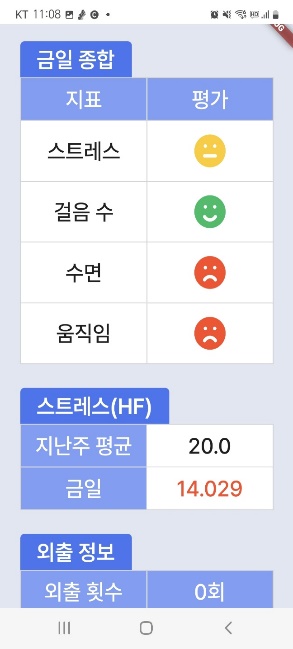 |
| --- | --- | --- | --- |
| 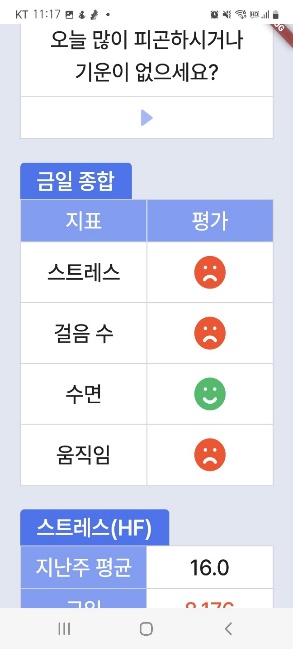 | 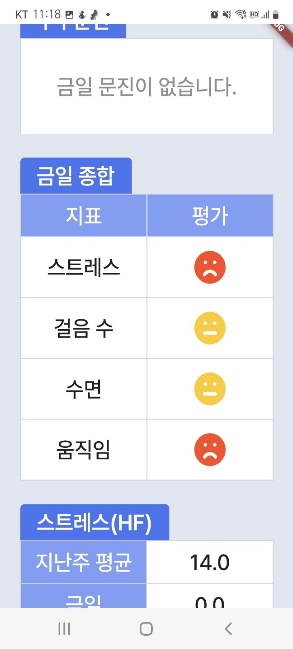 | 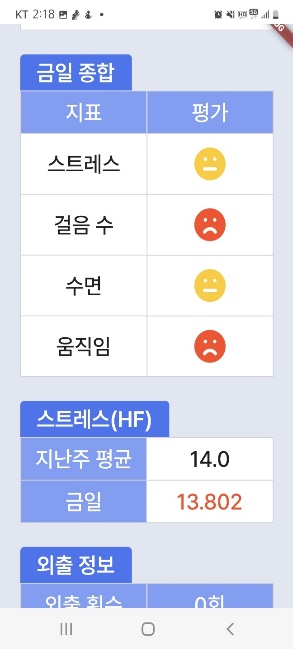 | 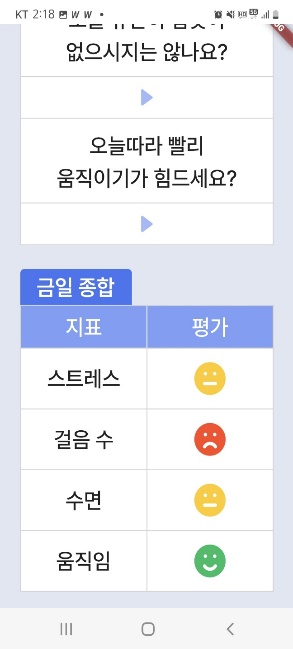 |
